# Chloroplast phylogenomic analysis of chlorophyte green algae identifies a novel lineage sister to the Sphaeropleales (Chlorophyceae)

**DOI:** 10.1186/s12862-015-0544-5

**Published:** 2015-12-01

**Authors:** Claude Lemieux, Antony T. Vincent, Aurélie Labarre, Christian Otis, Monique Turmel

**Affiliations:** Département de Biochimie, de Microbiologie et de Bio-informatique, Institut de Biologie Intégrative et des Systèmes, Université Laval, 1030 ave de la Médecine, Pavillon Marchand, Québec, QC G1V 0A6 Canada

**Keywords:** Chlorophyceae, Chlamydomonadales, Golenkiniaceae, *Treubarinia*, *Jenufa* genus, Plastid genome, Flagellar apparatus

## Abstract

**Background:**

The class Chlorophyceae (Chlorophyta) includes morphologically and ecologically diverse green algae. Most of the documented species belong to the clade formed by the Chlamydomonadales (also called Volvocales) and Sphaeropleales. Although studies based on the nuclear 18S rRNA gene or a few combined genes have shed light on the diversity and phylogenetic structure of the Chlamydomonadales, the positions of many of the monophyletic groups identified remain uncertain. Here, we used a chloroplast phylogenomic approach to delineate the relationships among these lineages.

**Results:**

To generate the analyzed amino acid and nucleotide data sets, we sequenced the chloroplast DNAs (cpDNAs) of 24 chlorophycean taxa; these included representatives from 16 of the 21 primary clades previously recognized in the Chlamydomonadales, two taxa from a coccoid lineage (*Jenufa*) that was suspected to be sister to the Golenkiniaceae, and two sphaeroplealeans. Using Bayesian and/or maximum likelihood inference methods, we analyzed an amino acid data set that was assembled from 69 cpDNA-encoded proteins of 73 core chlorophyte (including 33 chlorophyceans), as well as two nucleotide data sets that were generated from the 69 genes coding for these proteins and 29 RNA-coding genes. The protein and gene phylogenies were congruent and robustly resolved the branching order of most of the investigated lineages. Within the Chlamydomonadales, 22 taxa formed an assemblage of five major clades/lineages. The earliest-diverging clade displayed *Hafniomonas laevis* and the *Crucicarteria,* and was followed by the *Radicarteria* and then by the *Chloromonadinia*. The latter lineage was sister to two superclades, one consisting of the *Oogamochlamydinia* and *Reinhardtinia* and the other of the *Caudivolvoxa* and *Xenovolvoxa*. To our surprise, the *Jenufa* species and the two spine-bearing green algae belonging to the *Golenkinia* and *Treubaria* genera were recovered in a highly supported monophyletic group that also included three taxa representing distinct families of the Sphaeropleales (Bracteacoccaceae, Mychonastaceae, and Scenedesmaceae).

**Conclusions:**

Our phylogenomic study advances our knowledge regarding the circumscription and internal structure of the Chlamydomonadales, suggesting that a previously unrecognized lineage is sister to the Sphaeropleales. In addition, it offers new insights into the flagellar structures of the founding members of both the Chlamydomonadales and Sphaeropleales.

**Electronic supplementary material:**

The online version of this article (doi:10.1186/s12862-015-0544-5) contains supplementary material, which is available to authorized users.

## Background

The Chlorophyceae occupies the crown of the Chlorophyta, one of the two divisions of the Viridiplantae [1]. This monophyletic class of green algae comprises five orders or main clades [2, 3] that form two major lineages [4, 5]: the Chlamydomonadales (or Volvocales) + Sphaeropleales (CS clade) and the Oedogoniales + Chaetophorales + Chaetopeltidales (OCC clade). Members of the Chlorophyceae are found in a wide range of habitats and display diverse cell organizations (unicells, coccoids, colonies, simple flattened thalli, unbranched and branched filaments) [1, 6]. Their motile cells also exhibit variability at the level of the flagellar apparatus. The flagellar basal bodies of most chlorophycean green algae are displaced in a clockwise (CW, 1–7 o’clock) direction or are directly opposed (DO, 12–6 o’clock). In the Chlamydomonadales (often designated as the CW clade), biflagellates display a CW orientation of basal bodies, whereas quadriflagellates harbor distinct and more complex flagellar apparatus ultrastructures [7, 8]. The vegetatively nonmotile unicellular or colonial taxa found in the Sphaeropleales (DO clade) produce zoospores with two flagella arranged in a DO configuration [9]. Quadriflagellates with the perfect DO configuration of flagellar bodies characterize the Chaetopeltidales [10], whereas quadriflagellates from the Chaetophorales display a polymorphic arrangement in which one pair of basal bodies has the DO configuration and the other is slightly displaced in a clockwise orientation [11, 12]. The members of the Oedogoniales have an unusual flagellar apparatus that is characterized by a stephanokont arrangement of flagella [13].

The Chlamydomonadales, the largest order of the Chlorophyceae, contains about half of the 3,336 species currently described in this class [14]. Phylogenetic studies based on the nuclear 18S rRNA gene [7, 15–22] and/or a few chloroplast genes (e.g. *atpB*, *psaB*, *rbcL*) [23–25] as well as combined 18S and 26S rDNAs [26] have highlighted numerous chlamydomonadalean lineages. In an exhaustive phylogenetic analysis of the 449 chlamydomonadalean 18S rDNA sequences available in GenBank at the time, Nakada et al. [16] uncovered 21 primary clades following the rules of PhyloCode. The sequences of the spine-bearing *Golenkinia* species were used to root the phylogeny because a sister relationship between the Golenkiniaceae and the Chlamydomonadales had been reported earlier [27] and also because the motile cells of these two groups have a CW basal body configuration [28]. Nakada et al. [16] identified a sister relationship for the strongly supported *Xenovolvoxa* and *Caudivolvoxa* superclades, which are composed of four and six primary clades, respectively. All remaining clades were found to be basal relative to these superclades, with the four deepest-branching lineages displaying quadriflagellates (*Hafnionomas, Treubarinia*, *Radicarteria,* and *Crucicarteria* clades) as originally described by Nozaki et al. [24]. The interrelationships between most of the primary clades, however, received low statistical support and were influenced by the method used for phylogenetic inference. Phylogenetic studies based on a combination of two or three genes (*atpB, psaB, rbcL*, 18S and 26S rDNAs) yielded trees with improved statistical support for some of the clades, but their topologies were variable depending on the gene data sets employed and were generally in conflict with 18S rDNA phylogenies. One study based on 18S and 26S rDNAs even called into question the alliance of the deep-branching *Treubarinia* clade with the Chlamydomonadales [2].

In the present investigation, we used a chloroplast phylogenomic approach to resolve problematic relationships among the major chlamydomonadalean clades proposed by Nakada et al. [16]. Our taxon sampling included representatives from 16 of the 21 primary clades as well as two taxa from a coccoid lineage (*Jenufa*) that was suspected to be sister to the Golenkiniaceae [19]. We undertook the partial or complete sequencing of 24 chlorophycean chloroplast genomes in order to generate the analyzed amino acid and nucleotide data sets. The results of our phylogenomic analyses enabled us to resolve the branching order of most of the investigated clades. Unexpectedly, the two *Jenufa* species and the representatives of the Golenkiniaceae and *Treubarinia* were recovered in a highly supported monophyletic group that also included the taxa belonging to the Sphaeropleales.

## Results

The 24 chlorophycean taxa that were selected for chloroplast DNA (cpDNA) sequencing are listed in Table 1. As mentioned earlier, they represent 16 of the 21 primary clades proposed by Nakada et al. [16] and also include two *bona fide* sphaeroplealean taxa representing the Bracteacoccaceae and Mychonastaceae. When we undertook our study, the partial or complete cpDNA sequences of only five taxa from the CS clade were available: *Chlamydomonas reinhardtii* [29], *Volvox carteri* f. *nagariensis* [30], *Chlamydomonas moewusii* [5], *Dunaliella salina* [31] and *Scenedesmus obliquus* [32]. We used the Roche 454 or Illumina platform to sequence the chloroplast genomes of the examined taxa and obtained complete genome sequences for 13 taxa (Table 1). The contigs making up the assemblies of the remaining chloroplast genomes (7–111 contigs) typically exhibited repeats at their extremities and one or more genes in their internal portions, suggesting that the presence of abundant repeats in intergenic regions prevented the assembly of complete genome sequences. Despite this problem, most if not all genes were recovered from the chloroplast genome of each examined taxon. We present here our phylogenetic analyses of concatenated chloroplast genes and proteins; the salient features of the newly sequenced chloroplast genomes will be reported in a separate article.

**Table 1:** Chlorophycean taxa whose chloroplast genomes were sequenced in this study

Taxa	Source^a^	Clade	Accession no^b^	Sequencing method
Chlorophyceae *incertae sedis*				
* Jenufa minuta*	CAUP H8102		[GenBank:KT625414]*	Roche 454
* Jenufa perforata*	CAUP H8101		[GenBank:KT625413]*	Illumina
Sphaeropleales				
* Bracteacoccus giganteus*	UTEX 1251	Bracteacoccaceae	[GenBank:KT625421]*	Roche 454
* Mychonastes jurisii*	SAG 37.98	Mychonastaceae	[GenBank:KT625411]*	Roche 454
Chlamydomonadales^c^				
* Golenkinia longispicula*	SAG 73.80	*Golenkinia*	[GenBank:KT625092 - KT625150]	Roche 454
* Treubaria triappendiculata*	SAG 38.83	*Treubarinia*	[GenBank:KT625410]*	Roche 454
* Hafniomonas laevis*	NIES 257	*Hafniomonas*	[GenBank:KT625415]*	Roche 454
* Carteria cerasiformis*	NIES 425	*Crucicarteria*	[GenBank:KT625420]*	Roche 454
* Carteria crucifera*	UTEX 432	*Crucicarteria*	[GenBank:KT624870 - KT624932]	Roche 454
* Carteria* sp	SAG 8–5	*Radicarteria*	[GenBank:KT625419]*	Roche 454
* Chloromonas typhlos* ^d^	UTEX LB 1969	*Chloromonadinia*	[GenBank:KT624630 - KT624716]	Roche 454
* Chloromonas radiata*	UTEX 966	*Chloromonadinia*	[GenBank:KT625008 - KT625084]	Roche 454
* Oogamochlamys gigantea*	SAG 44.91	*Oogamochlamydinia*	[GenBank:KT625412]*	Illumina
* Lobochlamys segnis*	SAG 9.83	*Oogamochlamydinia*	[GenBank:KT624806 - KT624869]	Roche 454
* Lobochlamys culleus*	SAG 19.72	*Oogamochlamydinia*	[GenBank:KT625151 - KT625204]	Roche 454
* Chlamydomonas asymmetrica*	SAG 70.72	*Reinhardtinia*	[GenBank:KT624933 - KT625007]	Roche 454
* Phacotus lenticularis*	SAG 61–1	*Phacotinia*	[GenBank:KT625422]*	Illumina
* Microglena monadina* ^e^	SAG 31.72	*Monadinia*	[GenBank:KT624717 - KT624805]	Illumina
* Characiochloris acuminata*	SAG 31.95	*Characiosiphonia*	[GenBank:KT625418]*	Illumina
* Chlamydomonas applanata*	SAG 11–9	*Polytominia*	[GenBank:KT625417]*	Roche 454
* Stephanosphaera pluvialis*	SAG 78-1a	*Stephanosphaerinia*	[GenBank:KT625299 - KT625409]	Illumina
* Chloromonas perforata*	SAG 11–43	*Stephanosphaerinia*	[GenBank:KT625416]*	Illumina
* Haematococcus lacustris*	SAG 34-1b	*Chlorogonia*	[GenBank:KT625205 - KT625298]	Illumina
* Chlorogonium capillatum*	UTEX 11	*Chlorogonia*	[GenBank:KT625085 - KT625091]	Illumina

^a^The taxa originate from the culture collections of algae at the University of Goettingen (SAG, [67]), the University of Texas at Austin (UTEX, [68]), the National Institute of Environmental Studies in Tsukuba (NIES, [69]), and Charles University in Prague (CAUP, [70])

^b^The GenBank accession number of the chloroplast genome is given for each taxon. The asterisks denote the genomes that were completely sequenced

^c^The clade designation of the chlamydomonadelean taxa follows the PhyloCode classification scheme of Nakada et al. [16]

^d^Previously designated as *Chlamydomonas nivalis*

^e^Previously designated as *Chlamydomonas monadina*

### Phylogenomic analyses of 69 cpDNA-encoded proteins

We initiated our phylogenomic study by analyzing an amino acid data set (PCG-AA) that was assembled from 69 cpDNA-encoded proteins of 73 core chlorophytes (total of 14,209 sites; see Methods for the list of corresponding genes). Missing data were allowed but their proportion accounted for only 1.6 % of the total data set. This data set was analyzed with PhyloBayes using the site-heterogeneous CATGTR + Γ4 model and also with RAxML using the site-homogeneous GTR + Γ4 model. In the latter analysis, the data set was partitioned by gene, with the model applied to each partition.

**Fig. 1:**
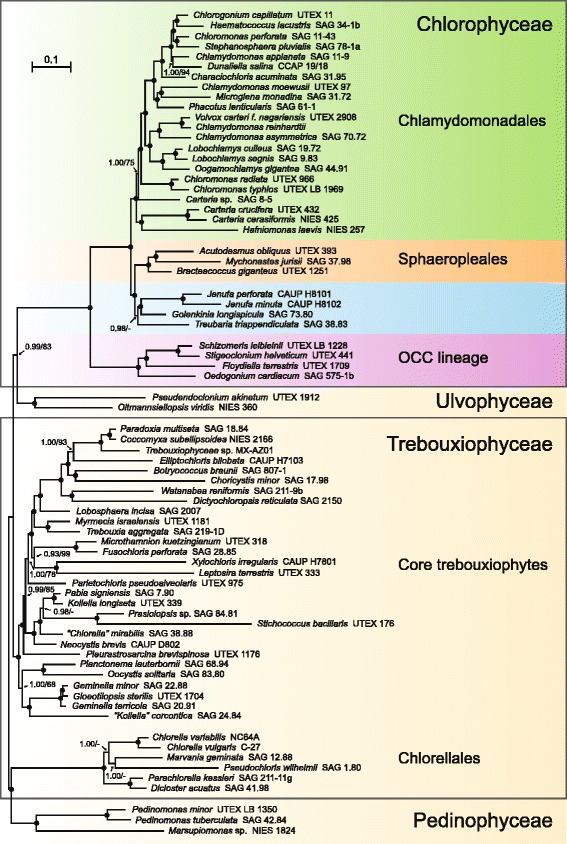
Phylogeny of 73 core chlorophytes inferred using the PCG-AA data set assembled from 69 cpDNA-encoded proteins. The tree presented here is the majority-rule posterior consensus tree inferred under the CATGTR + Γ4 model. Support values are reported on the nodes: from left to right, are shown the posterior probability (PP) values for the PhyloBayes CATGTR + Γ4 analyses and the BS values for the RAxML GTR + Γ4 analyses. Black dots indicate that the corresponding branches received PP values of 1.00 and BS values ≥ 95 % in the analyses; a dash denotes a BS value < 50 %. The scale bar denotes the estimated number of amino acid substitutions per site. Note that the genus *Pseudendoclonium* (Ulvophyceae) is polyphyletic and that *P. akinetum* (Ulotrichales), a close relative of *Trichosarcina* species, was wrongly classified in this genus [66]

The majority-rule consensus trees inferred using maximum likelihood (ML) and Bayesian inference methods display essentially the same topology, with high bootstrap support (BS) values found at most of the nodes (Fig. 1). As expected, the relationships observed for the pedinophyceans, trebouxiophyceans and ulvophyceans that were used as outgroup taxa are essentially identical to those reported by Lemieux et al. [33]. In addition, the strongly supported clade formed by the algae in the OCC lineage is sister to the strongly supported clade uniting the Chlamydomonadales and the Sphaeropleales (CS clade). The three representatives of recognized families within the Sphaeropleales (Bracteacoccaceae, Mychonastaceae, and Scenedesmaceae) form a robust clade. In the Bayesian tree, this clade occupies a sister position relative to that containing the two *Jenufa* species, *Golenkinia longispicula* and *Treubaria triappendiculata*. The node coinciding with the ancestor of these seven species received maximal support in both the Bayesian and ML analyses. However, *Treubaria* is positioned differently in these analyses: instead of being sister to the *Golenkinia* and *Jenufa* lineages as in the Bayesian tree, it branches at the base of the lineages containing *Bracteococcus giganteus*, *Mychonastes jurisii* and *Scenedesmus obliquus* in the ML tree (BS = 54 %).

The 22 taxa within the Chlamydomonadales form an assemblage of five major clades/lineages, all of which received very strong support in both the Bayesian and ML trees, with the exception of the lineage leading to *Carteria* sp. SAG 8–5 (BS = 75 %). The earliest-diverging clade, which includes *Hafniomonas laevis* and the *Crucicarteria* (*Carteria crucifera* and *Carteria cerasiformis*), is followed by the *Radicarteria* (*Carteria* sp. SAG 8–5). The next lineage, which is occupied by the representatives of the *Chloromonadinia* (two *Chloromonas* species), is sister to an assemblage formed by two major clades, one consisting of the *Oogamochlamydinia* (*Oogamochlamys* and two *Lobochlamys* species) and *Reinhardtinia* (*Volvox* and two *Chlamydomonas* species) and the other of the *Caudivolvoxa* and *Xenovolvoxa*. The *Caudivolvoxa* contains representatives of the *Characiosiphonia* (*Characiochloris*), *Chlorogonia* (*Chlorogonium* and *Haematococcus*), *Dunaliellinia* (*Dunaliella*), *Polytomia* (*Chlamydomonas applanata*) and *Stephanosphaerinia* (*Stephanosphaera* and *Chloromonas perforata*), while the *Xenovolvoxa* contains representatives of *Moewusinia* (*Chlamydomonas moewusii*), *Monadinia* (*Microglena*) and *Phacotinia* (*Phacotus*).

### Phylogenomic analyses of 98 chloroplast genes

**Fig. 2:**
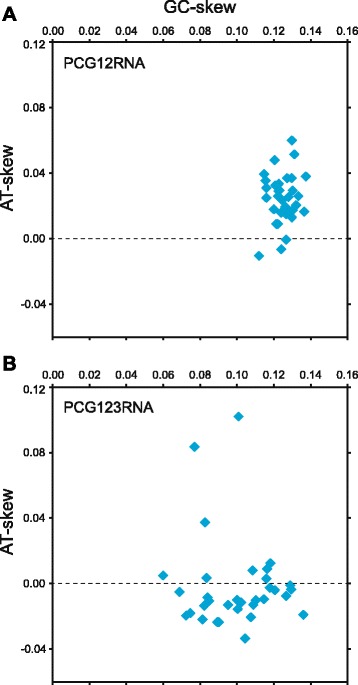
GC/AT-skew plots of the PCG12RNA (**a**) and PCG123RNA (**b**) nucleotide data sets. The nucleotide skew values were calculated using DAMBE [35]; each point corresponds to one of the 33 chlorophycean taxa included in the data sets

We also wished to infer trees using the 69 genes corresponding to the proteins represented in the amino acid data set as well as 29 RNA-coding genes (three rRNA genes and 26 tRNA genes). Before undertaking these analyses, we evaluated the phylogenetic performance of the third codon positions using the saturation test of Xia et al. [34]. We found that the index of substitution saturation (Iss = 0.624) was significantly higher (*P* < 0.001) than the critical value of the index of saturation (IssAsym = 0.573), implying that third codon positions experienced a high level of saturation and are thus useless for phylogenetic reconstructions. Furthermore, the AT- and GC-skew calculations carried out with DAMBE [35] using the PCG12RNA (34,121 sites) and PCG123RNA (48,172 sites) data sets, which differ only by the presence/absence of third codon positions, indicated that inclusion of the third codon positions induced nucleotide compositional bias. As shown in Fig. 2, the AT-skew values turned negative for many taxa in the data set containing these codon positions and both the AT- and GC-skew values became more scattered. To reduce the saturation level and compositional bias contributed by the third codon positions, we assembled the PCG123degenRNA data set (48,172 sites) in which all codon positions of the 69 protein-coding genes were fully degenerated using degen1 [36]. This script operates by degenerating nucleotides at all sites that can potentially undergo synonymous change in all pairwise comparisons of sequences in the data matrix, thereby making synonymous changes largely invisible and reducing compositional heterogeneity but leaving the inference of nonsynonymous changes largely intact.

The PCG12RNA and PCG123degenRNA data sets were analyzed with RAxML using the GTR + Γ4 model of sequence evolution. These data sets, which contained only 1.4 % of missing data, were partitioned into 71 groups with the model applied to each partition. The partitions included the 69 individual protein-coding genes, the concatenated rRNA genes and the concatenated tRNA genes. The inferred majority-rule consensus gene trees are essentially congruent with the protein trees (Additional file 1); moreover, the same lineages received weak support in both the gene and protein trees (i.e. the *Treubaria* and *Carteria* sp. SAG 8–5 lineages). The only notable deviation with the protein trees is that the affiliation of *Hafniomonas* with the *Crucicarteria* suffered from low statistical support in both nucleotide-based phylogenetic analyses. As observed in the protein trees, *Treubaria* is found either at the base of the clade containing *Golenkinia* and the two *Jenufa* species (in the tree inferred from the PCG12RNA data set) or at the base of the clade containing *Bracteococcus*, *Mychonastes* and *Scenedesmus* (in the tree inferred from the PCG123degenRNA).

## Discussion

**Fig. 3:**
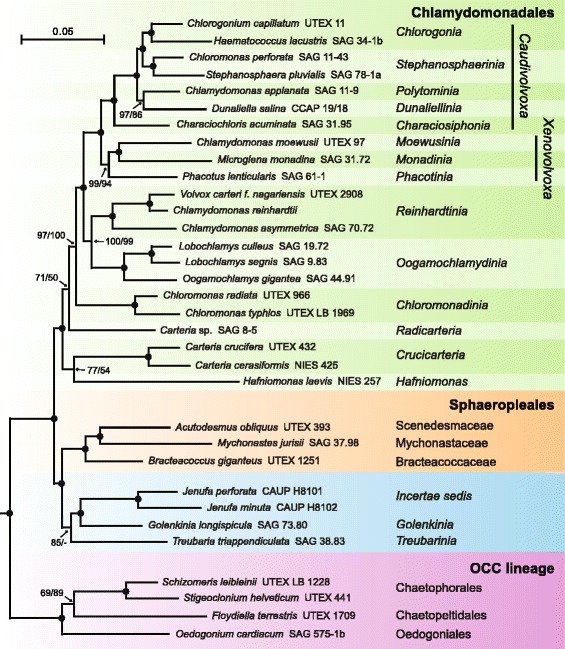
Phylogeny of chlorophycean taxa inferred using nucleotide data sets assembled from 69 protein-coding and 29 RNA-coding genes. The tree presented here is the best-scoring ML tree inferred using the PCG12RNA data set under the GTR + Γ4 model. Note that the portion of the tree containing the pedinophycean and trebouxiophycean outgroup taxa is not shown (see Additional file 1 for the complete topology). Support values are reported on the nodes: from left to right, are shown the BS values for the analyses of the PCG12RNA and PCG123degenRNA data sets. Black dots indicate that the corresponding branches received BS values of 100 % in the two analyses; a dash denotes a BS support lower than 50 %. Shaded areas identify the clades that are well supported in 18S rDNA phylogenies. The scale bar denotes the estimated number of nucleotide substitutions per site

In this study, we used a phylogenomic approach to resolve ambiguous nodes in the phylogeny of the Chlamydomonadales and to better delineate this chlorophycean order from its sister lineage, the Sphaeropleales. The chloroplast genome sequences we gathered from 24 taxa allowed us to increase taxon sampling for these chlorophycean lineages by a factor of 6-fold compared to earlier phylogenomic analyses. We examined a total of 29 chlamydomonadalean and sphaeroplealean taxa that represent 16 of the 21 primary clades that Nakada et al. [16] recovered for the Chlamydomonadales, three of the major lineages recognized for the Sphaeropleales (Scenedesmaceae, Mychonastaceae and Bracteacoccaceae) as well as the *Jenufa* lineage, which was suspected to be sister to the *Golenkinia* outgroup used by Nakada et al. [16]. The trees inferred from both the amino acid and nucleotide data sets received strong statistical support for the majority of branches (Figs. 1 and 3), providing important insights into the phylogenies of the Chlamydomonadales and Sphaeropleales.

### A newly identified lineage sister to the Sphaeropleales

Our results suggest that a previously unrecognized lineage of the Chlorophyceae is sister to the Sphaeropleales (Figs. 1 and 3). This lineage is detected as a strongly supported clade that unites the spine-bearing *Golenkinia longispicula* with the two *Jenufa* species (Figs. 1 and 3). This clade may also comprise the *Treubarinia*; the latter multi-genera lineage, which includes *Cylindrocapsa*, *Trochiscia* and *Elakatothrix* in addition to *Treubaria* [2, 16, 37], is represented in our study by the spine-bearing *Treubaria triappendiculata*. While the latter alga is sister to the *Golenkinia* + *Jenufa* lineages in the Bayesian protein tree (PP = 0.98) and the ML gene tree inferred with the PCG12RNA data set (BS = 85 %), it branches at the base of the lineages formed by the representatives of the Scenedesmaceae, Mychonastaceae and Bracteacoccace in the ML protein tree (BS = 54 %) and the gene tree inferred with the PCG123degenRNA data set (BS = 54 %). Like the *Golenkinia* species, some members of the *Treubarinia* (*Treubaria* and *Trochiscia* species) bear spines, but spine morphology and composition differ in the two lineages [28].

Among the previously published phylogenies of chlorophyceans, only the 18S + 28S rDNA study of Shoup and Lewis [37] recovered the *Treubarinia* within the Sphaeropleales. These authors sampled diverse lineages of the Chlamydomonadales and Sphaeropleales but no representatives of the *Golenkinia* were examined. In both the Bayesian and maximum parsimony trees they inferred, *Cylindrocapsa geminella* and *Trochiscia hystrix* were found to be sister to the Sphaeropleaceae, but support was weak (BS < 50 % and PP < 0.50). In contrast, the previously reported 18S + 28S rDNA analyses of Buchheim et al. [2] revealed, with weak support, an affinity between the *Treubaria* + *Cylindrocapsa* lineage and the Chlamydomonadales. More recently, consistent with the 18S rDNA trees inferred by Gerloff-Elias et al. [15], the 18S rDNA analyses of Němcová et al. [19] recovered the *Treubarinia* as sister to the *Hafniomonas* lineage (Chlamydomonadales) but again with no statistical support.

The taxonomic history of the *Golenkinia* genus is confusing because it underwent several revisions. Ettl and Komárek [38] included *Golenkinia* together with *Chlorotetraedron* and *Polyedriopsis* in the Neochloridaceae, a family of the Sphaeropleales containing aquatic coccoid algae that are mostly multinucleate. Subsequently, Komárek and Fott [39] erected the Golenkiniaceae to accommodate unicellular algae exhibiting spherical cells with spiny projections on their cells walls, including *Golenkinia* and *Polyedriopsis*. However, an affinity between these two genera could not be confirmed by 18S rDNA analyses: it was found that *Polyedriopsis* is closely related to members of the Neochloridaceae, in particular *Neochloris* and *Chorotetraedron* [40] but that *Golenkinia* is sister to the Chlamydomonadales [27, 41]. Using also the 18S rDNA marker, Němcová et al. [19] identified a loose relationship between the *Jenufa* and *Golenkinia* genera, which is in agreement with our study, but this clade could not be assigned to a specific chlorophycean order.

The novel lineage identified here as sister to the Sphaeropleales possibly represents one of the the deepest branch of this order. To elucidate its relationships to the major clades and families recognized in this order, phylogenomic analyses with a broader taxon sampling including all major sphaeroplealean lineages as well as additional representatives of the *Treubarinia* will be required. Fučíková et al. [42] recently proposed an updated family-level taxonomy comprising ten new families based on a study of seven genes (three nuclear and four chloroplast genes) from taxa sampled across the Sphaeropleales, but the relationships among most of the 17 recognized families could not be resolved. The inferred trees suggested that the genus *Mychonastes*, which contains aquatic uninucleate coccoid algae, may be the deepest-diverging lineage. This observation is not consistent with our finding that the soil multinucleate coccoid alga *Bracteococcus* (Bracteacoccaceae) is sister to the clade formed by the unicleate *Scenedesmus* (Scenedesmaceae) and *Mychonastes* (Mychonastaceae) (Figs. 1 and 3). The latter relationships, however, are compatible with the 18S rDNA tree of Němcová et al. [19].

### Relationships within the Chlamydomonadales

Prior to our investigation, multiple clades had been delineated for the Chlamydomonadales but their inter-relationships remained ambiguous. We present here for the first time a robust phylogeny of the Chlamydomonadales that resolves with confidence the branching order of most of the main lineages investigated (Figs. 1 and 3). In addition to the *Xenovolvoxa* and *Caudivolvoxa*, we recovered an additional superclade that unites the *Oogamochlamydinia* and *Reinhardtinia*; this superclade is sister to the *Caudivolvoxa* + *Xenovolvoxa*.

The internal structures of the *Caudivolvoxa* and *Xenovolvoxa* superclades were fully resolved in our trees. The topology observed for the *Caudivolvoxa* was identical to that found in the 18S rDNA phylogeny of Nakada et al. [16] and the three-gene phylogeny of Nakada and Tomita [18], but the clade containing the *Polytominia* and *Dunaliellinia* received little or no support in the latter phylogenies. As observed previously [16–19, 26], we found that the *Chlorogonia* and *Stephanosphaerinia* form a clade and that the *Characiosiphonia* is the most basal lineage of the *Caudivolvoxa*. For the *Xenovolvoxa*, we identified the *Phacotinia* as sister to the *Monadinia* + *Moewusinia* clade. In contrast, the three-gene phylogeny of Nakada and Tomita [18] and the 18S rDNA tree inferred by Nakada et al. [16] placed the *Moewusinia* at the base of this clade with little or no support.

The relationships we observed among the basal chlamydomonadalean lineages (*Crucicarteria* + *Hafniomonas*, *Radicarteria* and *Chloromonadinia*) were congruent with the three-gene (18S rDNA, *atpB* and *psaB*) phylogenies inferred by Nozaki et al. [25] and Matsuzaki et al. [23]. The representative of the *Radicarteria* (*Carteria* sp. SAG 8–5) was not positioned with high confidence in both our protein and gene trees and the sister-relationship between the *Hafniomonas* lineage and the *Crucicarteria* was poorly supported in the gene tree (Figs. 1 and 3). The *Tetraflagellochloris* and *Spermatozopsis* lineages, which were resolved as deep branches in 18S rDNA trees [7, 16, 17, 19, 22], will need to be sampled in future phylogenomic studies in order to clarify the branching order of the earliest-diverging lineages of the Chlamydomonadales. In the phylogeny inferred by Barsanti et al. [7], *Tetraflagellochloris mauritanica,* a quadriflagellate recently isolated from the desert, occupies the deepest position within this order and consistent with some other 18S rDNA studies [16, 17, 20], the *Spermatozopsis* lineage occupies a sister position relative to the *Radicarteria*; but, in contrast to our study and all other phylogenies reported so far, the *Hafniomonas* lineage was found to be allied with the *Reinhardtinia.*

### Evolution of flagellar apparatus structure

Considering that the novel clade reported here as sister to the Sphaeropleales contains at least one lineage with quadriflagellate motile cells (*Golenkinia*; *G. radiata*, the type species, is quadriflagellate while *G. longispicula* is biflagellate), it is reasonable to propose, as hypothesized by Nozaki et al. [24] for the Chlamydomonadales, that quadriflagellates also gave rise to the biflagellate motile cells found in the Sphaeropleales. Interestingly, the biflagellate motile cells of *G. longispicula* exhibits a CW orientation of basal bodies [27, 28], whereas all sphaeroplealean taxa that have been investigated for their flagellar apparatus have a DO configuration [9]. This phylogenetic distribution of flagellar architecture suggests that the quadriflagellate ancestor of these algae possessed a CW + DO organization and that loss of the flagellar pair exhibiting the CW organization gave rise to the DO flagellar apparatus in sphaeroplealeans. To substantiate this hypothesis, it would be important to examine the flagellar apparatus of quadriflagellate motile cells from genera belonging to both the *Golenkinia* and *Treubarinia*. In this connection, it is worth mentioning that two pairs of basal bodies with a unusual arrangement (diagonally opposed) have been reported for *Cylindrocapsa,* a member of the *Treubarinia* [43].

**Fig. 4:**
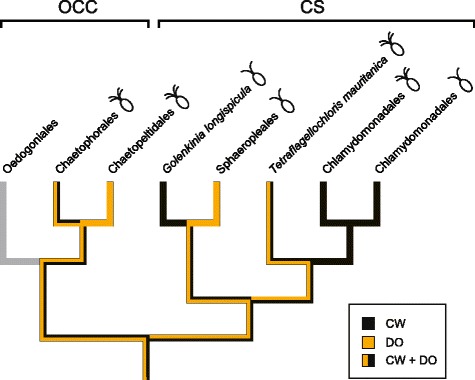
Evolution of the flagellar apparatus in the Chlorophyceae. The ancestral states of the absolute orientation of the flagellar apparatus were reconstructed using Mesquite 3.03 [64]. The most parsimonious scenario of character states is shown, with colored lines denoting the orientation patterns observed for biflagellate and quadriflagellate motile cells within the chlorophycean lineages. In the case of the Oedogoniales stephanokonts, the orientation pattern is ambiguous

Given the recent finding of a CW + DO flagellar architecture in the deeply branching chlamydomonadalean *Tetraflagellochloris mauritanica* [7], it appears that not only the architecture characteristic of the Sphaeropleales (DO) but also that characteristic of the Chlamydomonadales (CW) were derived from a CW + DO quadriflagellate ancestor. Actually, mapping of character states for the flagellar apparatus on the topology of the Chlorophyceae reveals that the quadriflagellate ancestor of all chlorophyceans also exhibited the CW + DO flagellar architecture (Fig. 4). In the predicted scenario, the DO condition changed to CW during the evolution of the Chlamydomonadales (CW + DO → CW + CW) and the CW condition changed to DO during the evolution of the Chaetopeltidales (CW + DO → DO + DO). The latter shift is not consistent with the hypothesis that O’Kelly and Floyd [44] proposed for the evolution of the flagellar apparatus in the green algae. According to this hypothesis, the CCW configuration displayed by the members of the Ulvophyceae and Trebouxiophyceae was converted to the CW configuration by progressive clockwise rotation of flagellar components, with the unidirectional sequence of arrangements proposed being CCW → DO → CW. The previous models based on phylogenetic and flagellar ultrastructural data were in agreement with the hypothesis of O’Kelly and Floyd [44]: the flagellar architecture of the ancestor of all chlorophyceans was the DO + DO configuration and convergent shifts from the DO to CW condition marked the evolution of the Chlamydomonadales and Chaetophorales [2, 5].

## Conclusions

Our chloroplast phylogenomic study advances our understanding regarding the circumscription of both the Chlamydomonadales and Sphaeropleales and the relationships of major lineages within these orders. We inferred robust protein and gene trees using the newly determined chloroplast genome sequences of 24 taxa representing 16 of the 21 primary clades previously recognized in the Chlamydomonadales and of two *Jenufa* species that belonged to a lineage of uncertain affiliation suspected to be sister to the Golenkiniaceae. Our most surprising discovery is the placement of the *Jenufa*, *Golenkinia* and *Treubaria* genera in a clade sister to the Sphaeropleales. Whether this new clade should be considered as part of the Sphaeropleales will await future analyses with a better representation of deep-branching lineages from both the Chlamydomonales and Sphaeropleales. Character state reconstruction of basal body orientation on the topology reported here for the Chlorophyceae also enabled us to refine the model for the evolution of the flagellar apparatus in this class.

The chloroplast genome sequences generated in this study constitute a highly valuable resource for future studies on the phylogeny of chlorophyceans and the evolution of their chloroplast genome. The 13 fully sequenced and annotated genomes more than double the number of chloroplast genomes publicly available for the Chlamydomonadales and Sphaeropleales. In a forthcoming article, we will show that these newly acquired sequences greatly improve our understanding of chloroplast genome evolution in the CS clade.

## Methods

### Strains and culture conditions

The 24 green algal strains that were selected for chloroplast genome sequencing are listed in Table 1. All of these strains, except *Carteria* sp. SAG 8–5, were obtained from the culture collections of algae at the University of Goettingen (SAG), the University of Texas at Austin (UTEX), the National Institute of Environmental Studies in Tsukuba (NIES), and Charles University in Prague (CAUP). *Carteria* sp. SAG 8–5 (=UTEX 2) was a gift of Dr. Mark Buchheim (University of Tulsa). All strains were grown in C medium [45] at 18 °C under alternating 12 h-light/12 h-dark periods.

### Genome sequencing, assembly and annotation

As indicated in Table 1, 15 of the genomes analyzed were sequenced using the Roche 454 method and the remaining nine using the Illumina method. For 454 sequencing, shotgun libraries (700-bp fragments) of A + T-rich DNA fractions obtained as described previously [46] were constructed using the GS-FLX Titanium Rapid Library Preparation Kit of Roche 454 Life Sciences (Branford, CT, USA). Library construction and 454 GS-FLX DNA Titanium pyrosequencing were carried out by the “Plateforme d’Analyses Génomiques de l’Université Laval” [47]. Reads were assembled using Newbler v2.5 [48] with default parameters, and contigs were visualized, linked and edited using the CONSED 22 package [49]. Contigs of chloroplast origin were identified by BLAST searches against a local database of organelle genomes. Regions spanning gaps in the cpDNA assemblies were amplified by polymerase chain reaction (PCR) with primers specific to the flanking sequences. Purified PCR products were sequenced using Sanger chemistry with the PRISM BigDye Terminator Ready Reaction Cycle Sequencing Kit (Applied Biosystems, Foster City, CA, USA).

For Illumina sequencing, total cellular DNA was isolated using the EZNA HP Plant Mini Kit of Omega Bio-Tek (Norcross, GA, USA). Libraries of 700-bp fragments were constructed using the TrueSeq DNA Sample Prep Kit (Illumina, San Diego, CA, USA) and paired-end reads were generated on the Illumina HiSeq 2000 (100-bp reads) or the MiSeq (300-bp reads) sequencing platforms by the Innovation Centre of McGill University and Genome Quebec [50] and the “Plateforme d’Analyses Génomiques de l’Université Laval” [47], respectively. Reads were assembled using Ray 2.3.1 [51] and contigs were visualized, linked and edited using the CONSED 22 package [49]. Identification of cpDNA contigs and gap filling were performed as described above for 454 sequence assemblies.

Genes were identified on the final assemblies using a custom-built suite of bioinformatics tools [52]. Genes coding for rRNAs and tRNAs were localized using RNAmmer [53] and tRNAscan-SE [54], respectively. Intron boundaries were determined by modeling intron secondary structures [55, 56] and by comparing intron-containing genes with intronless homologs.

### Phylogenomic analyses of the amino acid data set

The chloroplast genomes of 73 core chlorophyte taxa were used to generate the analyzed amino acid and nucleotide data sets. The GenBank accession numbers of the genomes sequenced in this study are presented in Table 1; those of the remaining genomes are as follows: *Pedinomonas minor* UTEX LB 1350, [GenBank:NC_016733]; *Pedinomonas tuberculata* SAG 42.84, [GenBank:KM462867]; *Marsupiomonas* sp. NIES 1824, [GenBank:KM462870]; *Pseudochloris wilhelmii* SAG 1.80, [GenBank:KM462886]; *Chlorella variabilis* NC64A, [GenBank:NC_015359]; *Chlorella vulgaris* C-27, [GenBank:NC_001865]; *Dicloster acuatus* SAG 41.98, [GenBank:KM462885]; *Marvania geminata* SAG 12.88, [GenBank:KM462888]; *Parachlorella kessleri* SAG 211-11 g, [GenBank:NC_012978]; *Botryococcus braunii* SAG 807–1, [GenBank:KM462884]; *Choricystis minor* SAG 17.98, [GenBank:KM462878]; *Coccomyxa subellipsoidea* NIES 2166, [GenBank:NC_015084]; *Elliptochloris bilobata* CAUP H7103, [GenBank:KM462887]; *Paradoxia multiseta* SAG 18.84, [GenBank:KM462879]; Trebouxiophyceae sp*.* MX-AZ01, [GenBank:NC_018569]; *Geminella minor* SAG 22.88, [GenBank:KM462883]; *Geminella terricola* SAG 20.91, [GenBank:KM462881]; *Gloeotilopsis sterilis* UTEX 1704, [GenBank:KM462877]; *Fusochloris perforata* SAG 28.85, [GenBank:KM462882]; *Microthamnion kuetzingianum* UTEX 318, [GenBank:KM462876]; *Oocystis solitaria* SAG 83.80, [GenBank:FJ968739]; *Planctonema lauterbornii* SAG 68.94, [GenBank:KM462880]; *“Chlorella” mirabilis* SAG 38.88, [GenBank:KM462865]; *Koliella longiseta* UTEX 339, [GenBank:KM462868]; *Pabia signiensis* SAG 7.90, [GenBank:KM462866]; *Stichococcus bacillaris* UTEX 176, [GenBank:KM462864]; *Prasiolopsis* sp. SAG 84.81, [GenBank:KM462862]; *Myrmecia israelensis* UTEX 1181, [GenBank:KM462861]; *Trebouxia aggregata* SAG 219-1D, [GenBank:EU123962-EU124002]; *Dictyochloropsis reticulata* SAG 2150, [GenBank:KM462860]; *Watanabea reniformis* SAG 211-9b, [GenBank:KM462863]; *Pleurastrosarcina brevispinosa* UTEX 1176, [GenBank:KM462875]; *“Koliella” corcontica* SAG 24.84, [GenBank:KM462874]; *Leptosira terrestris* UTEX 333, [GenBank:NC_009681]; *Lobosphaera incisa* SAG 2007, [GenBank:KM462871]; *Neocystis brevis* CAUP D802, [GenBank:KM462873]; *Parietochloris pseudoalveolaris* UTEX 975, [GenBank:KM462869]; *Xylochloris irregularis* CAUP H7801, [GenBank:KM462872]; *Oltmannsiellopsis viridis* NIES 360, [GenBank:NC_008099]; *Pseudendoclonium akinetum* UTEX 1912, [GenBank:NC_008114]; *Oedogonium cardiacum* SAG 575-1b, [GenBank:NC_011031]; *Floydiella terrestris* UTEX 1709, [GenBank:NC_014346]; *Stigeoclonium helveticum* UTEX 441, [GenBank:NC_008372]; *Schizomeris leibleinii* UTEX LB 1228, [GenBank:NC_015645]; *Scenedesmus obliquus* UTEX 393, [GenBank:NC_008101]; *Chlamydomonas moewusii* UTEX 97, [GenBank:EF587443-EF587503]; *Dunaliella salina* CCAP 19/18, [GenBank:NC_016732]; *Volvox carteri* f. *nagariensis* UTEX 2908, [GenBank:GU084820]; and *Chlamydomonas reinhardtii*, [GenBank:NC_005353].

A total of 69 protein-coding genes were used to construct the amino acid data set (PCG-AA): *atpA, B, E, F, H, I, ccsA, cemA, chlB, L, N, clpP, ftsH, infA, petA, B, D, G, L, psaA, B, C, J, M, psbA, B, C, D, E, F, H, I, J, K, L, M, N, T, Z, rbcL, rpl2, 5, 12, 14, 16, 20, 23, 32, 36, rpoA, B, C1, C2, rps2, 3, 4, 7, 8, 9, 11, 12, 14, 18, 19, tufA, ycf1, 3, 4, 12.* This data set was prepared as follows: the deduced amino acid sequences from the 69 individual genes were aligned using MUSCLE 3.7 [57], the ambiguously aligned regions in each alignment were removed using TRIMAL 1.3 [58] with the options block = 6, gt = 0.7, st = 0.005 and sw = 3, and the protein alignments were concatenated using Phyutility 2.2.6 [59].

Phylogenies were inferred from the PCG-AA data set using the ML and Bayesian methods. ML analyses were carried out using RAxML 8.1.14 [60] and the GTR + Γ4 model of sequence evolution; in these analyses, the data set was partitioned by gene, with the model applied to each partition. Confidence of branch points was estimated by fast-bootstrap analysis (f = a) with 500 replicates. Bayesian analyses were performed with PhyloBayes 3.3f [61] using the site-heterogeneous CATGTR + Γ4 model [62]. Five independent chains were run for 2,000 cycles and consensus topologies were calculated from the saved trees using the BPCOMP program of PhyloBayes after a burn-in of 500 cycles. Under these conditions, the largest discrepancy observed across all bipartitions in the consensus topologies (maxdiff) was lower than 0.15, indicating that convergence between the chains was achieved.

### Phylogenomic analyses of nucleotide data sets

Two DNA datasets were constructed: PCG123degenRNA (all degenerated codon positions of 69 protein-coding genes plus three rRNA genes and 26 tRNA genes) and PCG12RNA (first and second codon positions of the 69 protein-coding genes plus three rRNA genes and 26 tRNA genes). The PCG123degenRNA data set was prepared as follows. The multiple sequence alignment of each protein was converted into a codon alignment, the poorly aligned and divergent regions in each codon alignment were excluded using Gblocks 0.91b [63] with the -t = c, −b3 = 5, −b4 = 5 and -b5 = half options, and the individual gene alignments were concatenated using Phyutility 2.2.6 [59]. The Degen1.pl 1.2 script of Regier et al. [36] was applied to the resulting concatenated alignment (PCG123) and finally, the degenerated matrix was combined with the concatenated alignment of the following RNA genes: *rrf, rrl, rrs, trnA* (ugc), *C* (gca), *D* (guc), *E* (uuc), *F* (gaa), *G* (gcc), *G* (ucc), *H* (gug), *I* (cau), *I* (gau), *K* (uuu), *L* (uaa), *L* (uag), *Me* (cau), *Mf* (cau), *N* (guu), *P* (ugg), *Q* (uug), *R* (acg), *R* (ucu), *S* (gcu), *S* (uga), *T* (ugu), *V* (uac), *W* (cca), *Y* (gua). The latter genes were aligned using MUSCLE 3.7 [57], the ambiguously aligned regions in each alignment were removed using TRIMAL 1.3 [58] with the options block = 6, gt = 0.9, st = 0.4 and sw = 3, and the individual alignments were concatenated using Phyutility 2.2.6 [59]. To obtain the PCG12RNA data set, the third codon positions of the PCG123 alignment were excluded using Mesquite 3.03 [64] and the resulting alignment was merged with the filtered RNA gene alignment.

ML analyses of the PCG12RNA and PCG123degenRNA nucleotide data sets were carried out using RAxML 8.1.14 [60] and the GTR + Γ4 model of sequence evolution. In these analyses, the data sets were partitioned into 71 groups, with the model applied to each partition. The partitions included the 69 individual protein-coding genes, the concatenated rRNA genes and the concatenated tRNA genes. Confidence of branch points was estimated by fast-bootstrap analysis (f = a) with 500 replicates.

Nucleotide substitution saturation for each of the three codon positions of concatenated chlorophycean protein coding genes was assessed using the test of Xia et al. [34] implemented in DAMBE [35]. This program was also employed to calculate AT-skew and GC-skew of chlorophycean sequences within the PCG12RNA and PCG123RNA data sets as a measure of nucleotide compositional differences.

## Availability of supporting data

The sequence data generated in this study are available in GenBank under the accession numbers KT624630 - KT625422 (see Table 1). The data sets supporting the results of this article are available in the Dryad Digital Repository (doi: 10.5061/dryad.nh149) [65].

## Additional file

Additional file 1:
**Phylogeny of chlorophycean taxa inferred using nucleotide data sets assembled from 69 protein-coding and 29 RNA-coding genes.** The tree presented in this figure is the best-scoring ML tree inferred using the PCG12RNA data set under the GTR + Γ4 model. BS support values are reported for the ML analyses of the PCG12RNA and PCG123degenRNA data sets. (PDF 126 kb)

## Abbreviations

BS: bootstrap support
CCW: counterclockwise
cpDNA: chloroplast DNA
CS: Chlamydomonadales and Sphaeropleales
CW: clockwise
DO: directly opposed
OCC: Oedogoniales, Chaetophorales and Chaetopeltidales
PCR: polymerase chain reaction
PP: posterior probability
