# Supplementary material for: Chloroplast phylogenomic analysis of chlorophyte green algae identifies a novel lineage sister to the Sphaeropleales (Chlorophyceae)
Source: BMC Evol Biol. 2015 Dec 1;15:264. doi: 10.1186/s12862-015-0544-5 (PMC4665822; doi:10.1186/s12862-015-0544-5)
Supplement: Additional file 1: — Phylogeny of chlorophycean taxa inferred using nucleotide data sets assembled from 69 protein-coding and 29 RNA-coding genes. The tree presented in this figure is the best-scoring ML tree inferred using the PCG12RNA data set under the GTR + Γ4 model. BS support values are reported for the ML analyses of the PCG12RNA and PCG123degenRNA data sets. (PDF 126 kb) [file 12862_2015_544_MOESM1_ESM.pdf]

Additional file 1. Phylogeny of chlorophycean taxa inferred using nucleotide data sets assembled from 69 protein-coding and 29 RNA-coding genes.

The tree presented here is the best-scoring ML tree inferred using the PCG12RNA data set under the GTR+Γ4 model. Support values are reported on the nodes: from left to right, are shown the BS values for the analyses of the PCG12RNA and PCG123degenRNA data sets. Black dots indicate that the corresponding branches received BS values of 95% in the two analyses; a dash denotes a BS support lower than 50%. The scale bar denotes the estimated number of nucleotide substitutions per site.

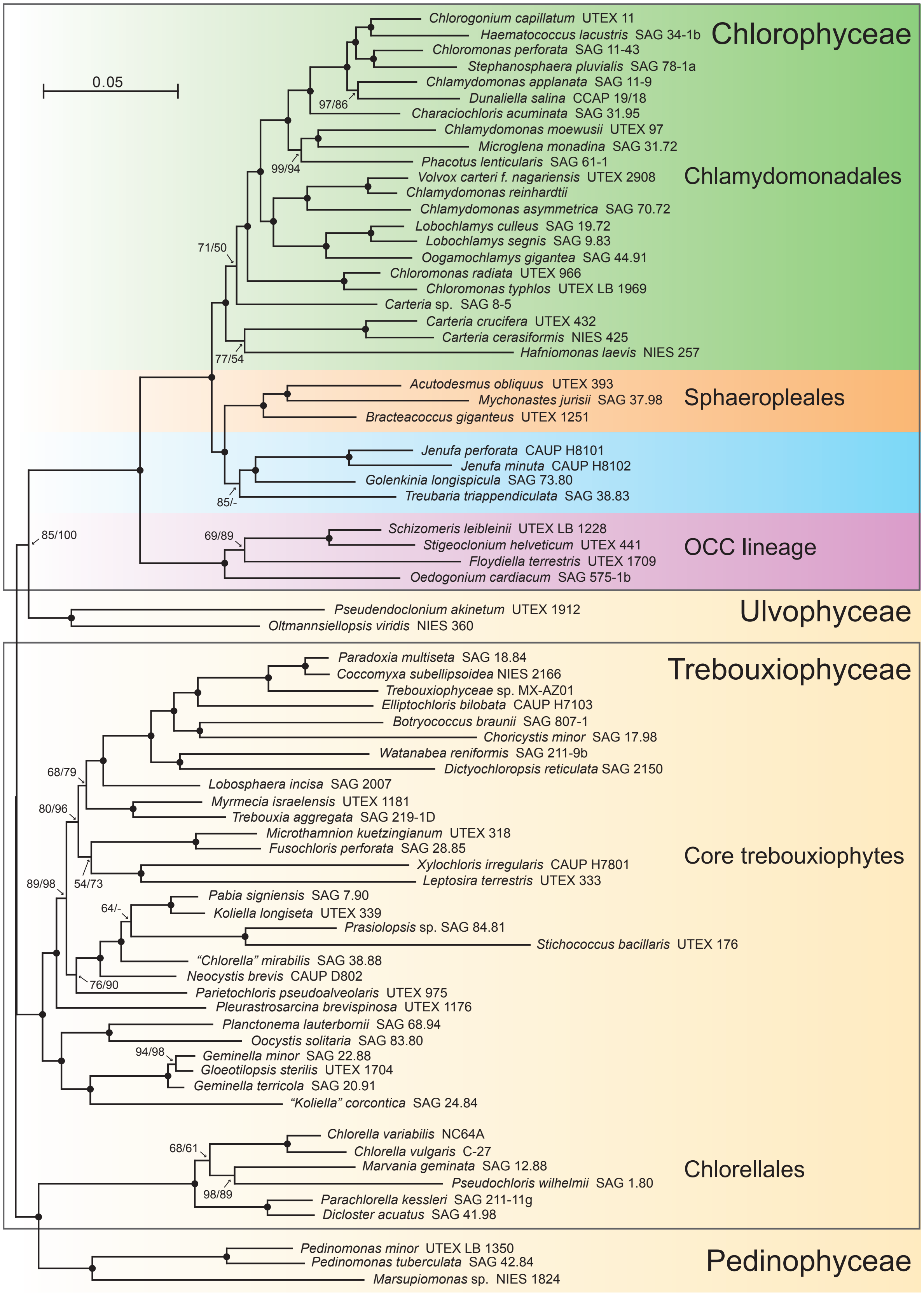

0.05
Chlorophyceae
Chlorogonium capillatum UTEX 11
Haematococcus lacustris SAG 34-1b
Chloromonas perforata SAG 11-43
Stephanosphaera pluvialis SAG 78-1a
Chlamydomonas applanata SAG 11-9
Dunaliella salina CCAP 19/18
Characiochloris acuminata SAG 31.95
Chlamydomonas moewusii UTEX 97
Microglena monadina SAG 31.72
Phacotus lenticularis SAG 61-1
Volvox carteri f. nagariensis UTEX 2908
Chlamydomonas reinhardtii
Chlamydomonas asymmetrica SAG 70.72
Lobochlamys culleus SAG 19.72
Lobochlamys segnis SAG 9.83
Oogamochlamys gigantea SAG 44.91
Chloromonas radiata UTEX 966
Chloromonas typhlos UTEX LB 1969
Carteria sp. SAG 8-5
Carteria crucifera UTEX 432
Carteria cerasiformis NIES 425
Hafniomonas laevis NIES 257
Chlamydomonadales
97/86
99/94
71/50
77/54
Acutodesmus obliquus UTEX 393
Mychonastes jurisii SAG 37.98
Bracteacoccus giganteus UTEX 1251
Sphaeropleales
Jenufa perforata CAUP H8101
Jenufa minuta CAUP H8102
Golenkinia longispicula SAG 73.80
Treubaria triappendiculata SAG 38.83
85/-
85/100
69/89
Schizomeris leibleinii UTEX LB 1228
Stigeoclonium helveticum UTEX 441
Floydiella terrestris UTEX 1709
Oedogonium cardiacum SAG 575-1b
OCC lineage
Pseudendoclonium akinetum UTEX 1912
Oltmannsiellopsis viridis NIES 360
Ulvophyceae
Trebouxiophyceae
Paradoxia multiseta SAG 18.84
Coccomyxa subellipsoidea NIES 2166
Trebouxiophyceae sp. MX-AZ01
Elliptochloris bilobata CAUP H7103
Botryococcus braunii SAG 807-1
Choricystis minor SAG 17.98
Watanabea reniformis SAG 211-9b
Dictyochloropsis reticulata SAG 2150
Lobosphaera incisa SAG 2007
Myrmecia israelensis UTEX 1181
Trebouxia aggregata SAG 219-1D
Microthamnion kuetzingianum UTEX 318
Fusochloris perforata SAG 28.85
Xylochloris irregularis CAUP H7801
Leptosira terrestris UTEX 333
Core trebouxiophytes
68/79
80/96
54/73
89/98
64/-
Pabia signiensis SAG 7.90
Koliella longiseta UTEX 339
Prasiolopsis sp. SAG 84.81
Stichococcus bacillaris UTEX 176
"Chlorella" mirabilis SAG 38.88
Neocystis brevis CAUP D802
76/90
Parietochloris pseudoalveolaris UTEX 975
Pleurastrosarcina brevispinosa UTEX 1176
Planctonema lauterbornii SAG 68.94
Oocystis solitaria SAG 83.80
94/98
Geminella minor SAG 22.88
Gloeotilopsis sterilis UTEX 1704
Geminella terricola SAG 20.91
"Koliella" corcontica SAG 24.84
68/61
Chlorella variabilis NC64A
Chlorella vulgaris C-27
Marvania geminata SAG 12.88
Pseudochloris wilhelmii SAG 1.80
98/89
Parachlorella kessleri SAG 211-11g
Dicloster acuatus SAG 41.98
Chlorellales
Pedinomonas minor UTEX LB 1350
Pedinomonas tuberculata SAG 42.84
Marsupiomonas sp. NIES 1824
Pedinophyceae
